# Genome-Wide Identification and Analysis of Expression Profiles of Maize Mitogen-Activated Protein Kinase Kinase Kinase

**DOI:** 10.1371/journal.pone.0057714

**Published:** 2013-02-27

**Authors:** Xiangpei Kong, Wei Lv, Dan Zhang, Shanshan Jiang, Shizhong Zhang, Dequan Li

**Affiliations:** 1 State Key Laboratory of Crop Biology, Shandong Key Laboratory of Crop Biology, College of Life Sciences, Shandong Agricultural University, Tai’an, Shandong, China; 2 State Key Laboratory of Crop Biology, Shandong Key Laboratory of Crop Biology, College of Horticulture Science and Engineering, Shandong Agricultural University, Tai’an, Shandong, China; National Taiwan University, Taiwan

## Abstract

Mitogen-activated protein kinase (MAPK) cascades are highly conserved signal transduction model in animals, yeast and plants. Plant MAPK cascades have been implicated in development and stress responses. Although MAPKKKs have been investigated in several plant species including Arabidopsis and rice, no systematic analysis has been conducted in maize. In this study, we performed a bioinformatics analysis of the entire maize genome and identified 74 MAPKKK genes. Phylogenetic analyses of MAPKKKs from maize, rice and Arabidopsis have classified them into three subgroups, which included Raf, ZIK and MEKK. Evolutionary relationships within subfamilies were also supported by exon-intron organizations and the conserved protein motifs. Further expression analysis of the MAPKKKs in microarray databases revealed that MAPKKKs were involved in important signaling pathways in maize different organs and developmental stages. Our genomics analysis of maize MAPKKK genes provides important information for evolutionary and functional characterization of this family in maize.

## Introduction

Mitogen-activated protein kinase (MAPK) cascades are conserved signal transduction pathways to translate external stimuli into cellular responses in all eukaryotes [1]. A typical MAPK cascade consists of three sequentially activated kinases. Upstream signals activate MAPK kinase kinases (MAPKKK or MEKK) which in turn phosphorylate a MAPK kinase (MKK or MEK); Subsequently, MKKs activate a specific MAPK. The downstream targets of MAPKs can be transcription factors or cytoskeletal proteins [2]–[5]. In plants, different MAPK pathways can be activated in various signaling pathways, including development, cell division, hormone responses, plant innate immunity, and abiotic stress [6]–[23].

The genome of *Arabidopsis* contains approximate 80 MAPKKKs, 10 MKKs, and 20 MAPKs [2]. Completion of the rice genome project has identified the existence of 75 MAPKKKs, 8 MKKs and 17 MAPKs [12], [24]. MAPKKKs act at the top of MAPK cascades and show great sequence diversity. In plants, MAPKKKs are divided into three groups according to their sequence alignment: the MEKK-like family, Raf-like family and ZIK-like family [2]. Compared with MAPKs and MKKs, plant MAPKKKs contain long N- or C- terminal regions that might function in scaffolding to recruit MKKs and MAPKs [20]. In *Arabidopsis*, several MAPKKKs have been involved in defense, ethylene signaling and plant development.

MEKK1-MKK4/5-MPK3/6-WRKY22/WRKY29 plays an important role in plant innate immunity [25]. MEKK1-MKK1/MKK2–MPK4 has previously been shown to play important roles in oxidative stress signaling, salt and cold stresses, whereas negatively regulates plant innate immunity [26]–[29]. More recently, Kong et al. (2012) and Zhang et al. (2012) identified that SUMM1 (MEKK2) functions as a positive regulator of the R protein SUMM2, and its activity is negatively regulated by the MEKK1-MKK1/2-MPK4 cascade [30], [31]. Recent genetic evidence has indicated that YDA–MKK4/MKK5–MPK3/MPK6 negatively regulates stomatal development through phosphorylation SPEECHLESS (SPCH) [17], [32]. CTR1 is able to inhibit MKK9–MPK3/MPK6 activation in ethylene signaling and probably acts as an unconventional MAPKKK [33], [34]. ANP2/3-MKK6-MPK4/11/13 plays roles in the regulation of cytokinesis [35]–[39]. In addition, Gao and Xiang (2008) reported that At1g73660 (AtRaf5) mutant exhibited an enhanced tolerance to salt in *Arabidopsis* [40]. In rice, overexpression *DSM1* (*OsMAPKKK6*) increased the tolerance to dehydration stress due to ROS scavenging [41]. Another Raf-like MAPKKK ILA1 (*OsMAPKKK43*) was identified involved in mechanical tissue formation in the leaf lamina joint in rice [42].

Maize (*Zea mays* L.) is one of the oldest and most important world-wide crops that are relied upon for human food, animal feed and for starch ethanol production. So far, seven MAPKs and 4 MKKs have been characterized in maize [43]–[51]. However, to our knowledge, the maize MAPKKK gene family has not been characterized in detail. In this study, we performed a bioinformatics analysis of the entire maize genome and identified 74 MAPKKK genes. In addition, we provide detailed information on the genomic structures, chromosomal locations and phylogenetic tree of maize MAPKKK genes. Subsequently, we investigated their transcript profiles in different organs and developmental stages using microarray data, which will help future studies for elucidating the precise roles of MAPKKKs in maize growth and development.

## Materials and Methods

### Identification of MAPKKK Gene Family in Maize

The completed genome sequence of *Zea mays* was downloaded from the maize sequence database (http://www.maizesequence.org/index.html). For the identification of maize MAPKKK gene family, Arabidopsis and rice MAPKKK protein sequences were firstly used as query sequences to search against the maize genome database and NCBI using BLASTP program. And self BLAST of the sequences was carried out to remove the redundancy. The Pfam (http://pfam.sanger.ac.uk/search) and SMART (http://smart.embl-heidelberg.de/) databases were used to confirm each predicted maize MAPKKK protein sequence.

### Gene Structure Analysis of Maize MAPKKK Genes

The information of maize MAPKKK genes, including accession number, chromosomal location, ORF length, exon-intron structure, were retrieved from the B73 maize sequencing database (http://www.maizesequence.org/index.html).

### Phylogenetic Analysis of Maize MAPKKK Proteins

Multiple alignments of MAPKKK proteins were carried out using the Clustal X v1.83 program. The protein sequences of Arabidopsis and rice MAPKKK were obtained from the TIGR database and phylogenetic analysis was performed with MEGA5.0 program by neighbor-joining method and the bootstrap test was carried out with 1000 replicates.

### Chromosomal Locations and Gene Duplication of MAPKKK Genes

Genes were mapped on chromosomes by identifying their chromosomal position provided in the maize sequence database. Gene duplication events of MAPKKK genes in maize B73 were also investigated. We defined the gene duplication in accordance with the criteria: 1) the alignment length covered >80% of the longer gene; 2) the aligned region had an identity >80%; 3) only one duplication event was counted for tightly linked genes. All of the relevant genes identified in the maize genomes were aligned using Clustal X v1.83 and calculated using MEGA v5.0.

### Expression Analyses of the MAPKKK Genes

Microarray expression data from various datasets were obtained making use of Genevestigator (https://www.genevestigator.com/gv/) with the Maize Gene Chip platform. The maize MAPKKK expression data was obtained through searching the Maize Gene Chip using identified MAPKKK ID (Table 1).

**Table 1:** Characteristics of MAPKKKs from maize.

Name	ID	Chr	cDNA	Amino acid	MW (kDa)	pI
MAPKKK1	GRMZM2G140726-T01	10	2879	727	78.3	9.22
MAPKKK2	GRMZM2G540772-T01	5	2173	600	66.9	9.56
MAPKKK3	BT034005.1^a^	2	2234	604	65.6	9.17
MAPKKK4	GRMZM2G175504-T01	2	2981	887	96.4	9.72
MAPKKK5	GRMZM2G093316-T01	4	3836	895	97.3	9.70
MAPKKK6	AC209208.3-FGT001	5	2967	988	107.6	9.70
MAPKKK7	GRMZM2G378479-T01	2	3014	742	81.4	9.55
MAPKKK8	GRMZM2G034877-T01	5	2070	689	75.0	9.72
MAPKKK9	GRMZM2G156800-T01	1	3397	755	81.7	9.39
MAPKKK10	GRMZM2G180555-T01	9	2374	599	65.1	6.31
MAPKKK11	GRMZM2G066120-T01	1	2327	600	65.4	6.37
MAPKKK12	GRMZM2G130927-T01	5	2444	629	67.4	5.44
MAPKKK13	GRMZM2G044557-T01	1	2443	633	67.9	5.23
MAPKKK14	GRMZM2G017654-T01	2	4440	1337	148.1	6.03
MAPKKK15	GRMZM2G064613-T01	4	2703	689	75.6	5.76
MAPKKK16	GRMZM2G098828-T01	2	2661	674	73.5	5.93
MAPKKK17	GRMZM2G439350-T01	8	1439	456	46.3	5.05
MAPKKK18	GRMZM2G305066-T01	8	1440	479	50.2	4.81
MAPKKK19	GRMZM2G165099-T01	3	1707	475	50.0	5.20
MAPKKK20	GRMZM2G476477-T01	6	1628	483	50.1	4.65
MAPKKK21	GRMZM2G173965-T01	8	1521	472	49.5	6.41
MAPKKK22	GRMZM2G041774-T01	3	1636	514	54.3	6.58
MAPKKK23	GRMZM2G116376-T01	4	1934	451	50.2	5.79
MAPKKK24	GRMZM5G878530-T03	5	2528	610	68.1	5.04
MAPKKK25	GRMZM2G084791-T01	4	2644	565	62.9	4.98
MAPKKK26	GRMZM2G021416-T02	7	2398	566	61.9	5.60
MAPKKK27	GRMZM2G089159-T01	2	2715	703	79.0	5.58
MAPKKK28	GRMZM2G312970-T01	6	2569	570	64.1	4.92
MAPKKK29	GRMZM2G011070-T01	0^b^	4285	1221	135.1	5.44
MAPKKK30	GRMZM2G326472-T03	9	4064	1114	119.5	5.60
MAPKKK31	GRMZM2G481005-T02	9	4027	1265	135.3	6.09
MAPKKK32	GRMZM2G039106-T01	4	4018	1139	126.2	5.49
MAPKKK33	GRMZM2G052658-T01	5	4087	1104	122.5	5.60
MAPKKK34	GRMZM2G038982-T01	1	3411	1136	122.9	6.87
MAPKKK35	GRMZM2G175563-T01	9	3009	892	98.0	6.11
MAPKKK36	GRMZM2G413069-T01	4	3310	869	94.5	5.35
MAPKKK37	GRMZM2G048243-T01	9	3915	1071	117.5	5.18
MAPKKK38	GRMZM2G098187-T04	2	2757	762	82.5	8.02
MAPKKK39	GRMZM2G059671-T05	5	2787	800	87.4	6.13
MAPKKK40	GRMZM2G110572-T02	1	3128	752	83.3	7.34
MAPKKK41	GRMZM2G140537-T01	3	3220	825	90.5	8.89
MAPKKK42	GRMZM2G448213-T01	4	2928	675	75.8	6.50
MAPKKK43	GRMZM2G007854-T01	4	2992	787	87.7	6.40
MAPKKK44	GRMZM2G163141-T03	8	3038	791	88.1	6.44
MAPKKK45	GRMZM2G080499-T01	3	3286	792	88.1	5.94
MAPKKK46	GRMZM2G159034-T01	7	1845	440	49.1	6.73
MAPKKK47	GRMZM2G045366-T01	3	2046	471	52.8	6.69
MAPKKK48	GRMZM5G882078-T02	3	1924	514	57.5	9.53
MAPKKK49	GRMZM2G007466-T02	5	2137	481	53.5	8.88
MAPKKK50	GRMZM2G111269-T01	1	2364	378	41.6	8.19
MAPKKK51	GRMZM2G019434-T01	1	1882	370	41.1	6.98
MAPKKK52	GRMZM2G131629-T01	1	1662	416	45.6	8.92
MAPKKK53	GRMZM2G014618-T01	5	1329	442	48.0	8.82
MAPKKK54	GRMZM2G063684-T02	8	1635	382	42.4	7.95
MAPKKK55	GRMZM2G088299-T01	3	1765	382	42.3	7.53
MAPKKK56	GRMZM2G063069-T01	8	1782	377	41.9	8.23
MAPKKK57	GRMZM2G165231-T01	4	1750	353	39.8	7.18
MAPKKK58	GRMZM5G814851-T01	7	2000	594	65.8	5.63
MAPKKK59	GRMZM2G459854-T01	2	2291	593	65.7	5.80
MAPKKK60	GRMZM2G164242-T02	5	2082	569	63.7	6.11
MAPKKK61	GRMZM2G160922-T02	7	2045	531	59.5	6.16
MAPKKK62	GRMZM2G465833-T01	10	2263	529	58.0	5.25
MAPKKK63	GRMZM2G152889-T01	3	1972	525	57.6	5.07
MAPKKK64	GRMZM2G156013-T01	10	2015	415	46.0	7.2
MAPKKK65	GRMZM2G102088-T01	2	3265	415	46.1	6.67
MAPKKK66	GRMZM2G140612-T01	10	2151	423	46.5	6.64
MAPKKK67	GRMZM2G028604-T01	9	2145	396	44.8	9.27
MAPKKK68	GRMZM2G018280-T01	6	2508	404	45.2	9.30
MAPKKK69	GRMZM2G171677-T01	3	2174	368	41.0	9.01
MAPKKK70	GRMZM2G097878-T01	8	2668	561	63.5	9.59
MAPKKK71	GRMZM2G055334-T01	3	2896	574	64.3	9.46
MAPKKK72	GRMZM2G114093-T01	1	2898	598	66.1	9.17
MAPKKK73	GRMZM2G474546-T03	6	1913	593	66.1	9.61
MAPKKK74	GRMZM2G104283-T01	8	2422	602	67.1	9.49

a GenBank accession numbers;

b unknown.

### Plant Materials and Growth Conditions

For maize inbred line Qi 319 (from Shandong Academy of Agricultural Sciences), embryo of 25 days after pollination was harvested from greenhouse-grown plants in sand under 16 h of light (25°C) and 8 h of dark (20°C), and eight-week-old seedling tissues and organs were harvested for expression analysis. Samples were collected and were immediately frozen in liquid N_2_ for further use. Two biological replicates were performed for each sample.

### RNA Isolation and Real-time Quantitative RT-PCR Expression Analysis

Total RNAs were extracted according to the instructions of Trizol reagent (Invitrogen, Carlsbad, CA, USA) from leaves of maize seedlings with different treatments. The first strand cDNAs were synthesized using First Strand cDNA Synthesis kit (Fermentas, USA).

Real-time quantification RT-PCR reactions were performed in Bio-RAD MyiQ™ Real-time PCR Detection System (Bio-Rad, USA) using the TransStart Top Green qPCR SuperMix (TransGen, China) according to the manufacturer’s instructions. Each PCR reaction (20 µl) contained 10 µl 2×real-time PCR Mix (containing SYBR Green I), 0.5 µl of each primer, and appropriately diluted cDNA. The thermal cycling conditions were 95°C for 30 s followed by 45 cycles of 95°C for 15 s, 55°C −60°C for 30 s, and 72°C for 15 s. The *Zmactin* gene was used as internal reference for all the qRT–PCR analysis. Each treatment was repeated three times independently. Relative gene expression was calculated according to the delta-delta Ct method of the system. The primers used are described in Table S1 in File S1.

## Results and Discussion

### Genome-wide Identification of MAPKKK Family in Maize

**Figure 1:**
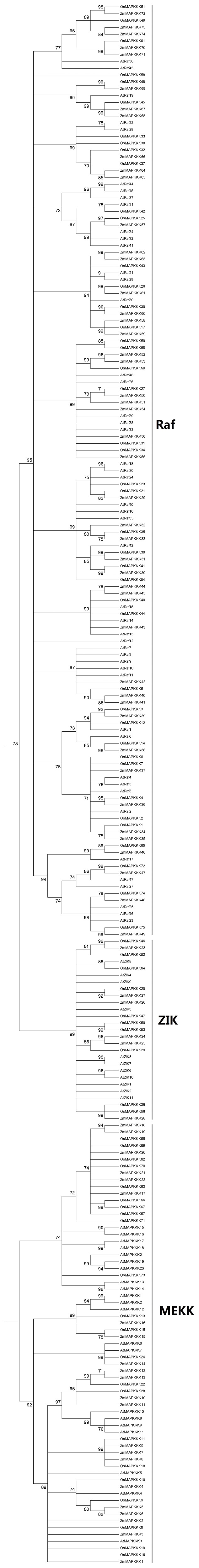
Phylogenetic tree of MAPKKKs from maize, rice and Arabidopsis. Neighbor-joining tree was created using MEGA5.0 program with 1,000 bootstrap using full length sequences of 74 maize, 75 rice, and 80 Arabidopsis MAPKKK proteins.

Availability of complete maize genome sequences has made it possible for the first time to identify all the MAPKKK family members in this plant species. BLAST searches of the maize sequences database and NCBI database were performed using 80 Arabidopsis and 75 rice MAPKKK sequences as query and this analysis has identified 74 putative MAPKKK gene family members in the complete maize genome, designated as ZmMAPKKK1-ZmMAPKKK74 according to their group, since there was no standard nomenclature followed for MAPKKKs neither in Arabidopsis nor in rice. All the 74 MAPKKKs had conserved protein kinase domains. Because there were alternative splice variants in some genes of the family, the following analysis was restricted to only a single variant for further analysis. The detailed information of maize MAPKKK genes identified in the present study, including accession numbers, number of amino acids, molecular weight, and isoelectric point (pI), was listed in Table 1. *ZmMAPKKK* ORF lengths ranged from 1062 bp (*ZmMAPKKK57*) to 4014 bp (*ZmMAPKKK14*) and the molecular weights ranged from 39.8 kDa (*ZmMAPKKK57*) to 148.1 kDa (*ZmMAPKKK14*). Since the size of maize genome (∼2300 Mb) is much larger than the genomes of Arabidopsis (125 Mb) and rice (389 Mb), MAPKKK genes in maize would be larger than that in Arabidopsis and rice. However, according to the present study, the number of maize MAPKKK genes was even smaller than that of *Arabidopsis* and rice (Figure 1).

### Comparative Phylogenetic Analysis of MAPKKK Gene in Maize, Arabidopsis and Rice

To examine the evolutionary relationships between different MAPKKK members in maize, Arabidopsis and rice, an unrooted tree was constructed from alignments of the full MAPKKK amino acid sequences using Neighbor-Joining (NJ) method by MEGA5.0 and phylogenetic analysis indicated that ZmMAPKKKs can be divided into three major groups: MEKK, Raf and ZIK. There were 46 MAPKKKs from maize, 43 from rice and 48 from Arabidopsis in Raf group. MEKK group contained 22 maize MAPKKKs, 22 rice MAPKKKs and 21 Arabidopsis MAPKKKs. Only 6 MAPKKKs from maize, 10 from rice and 11 from Arabidopsis were grouped into ZIK group (Figure 1).

The inspection of the phylogenetic tree indicated 19 *ZmMAPKKK* paralogous gene pairs and these gene pairs represented 52% of the maize MAPKKK genes family members (Figure S1 in File S1), suggesting maize *MAPKKK* gene family may have undergone multiple duplications during the evolution history. Phylogenetic analysis also showed that there were 16 pairs of maize/rice MAPKKK proteins in the same clade of the phylogenetic tree (Figure 1).

### Gene Structural Organization and Analysis of Conserved Domain in MAPKKK Genes

**Figure 2:**
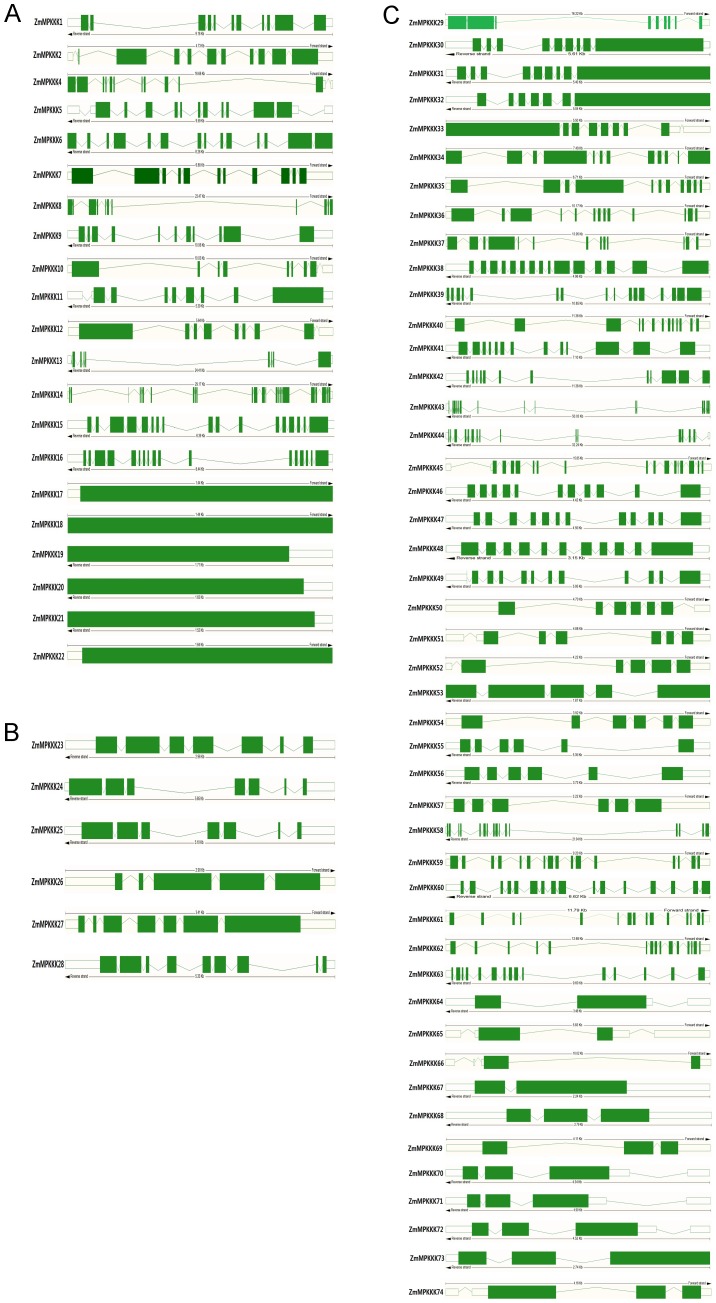
Exon–intron structures of maize *MAPKKK* genes. Boxes, exons; green boxes, open reading frames; lines, introns. A, MEKK; B, ZIK; C, Raf.

Based on the predicted sequences, the maize MAPKKK gene structures were determined. As shown in Figure 2, there were 8–17 exons in most maize MEKK group genes, whereas six genes (*ZmMAPKKK17*, *ZmMAPKKK18*, *ZmMAPKKK19*, *ZmMAPKKK20*, *ZmMAPKKK21* and *ZmMAPKKK22*) only had one exon, and one gene (*ZmMAPKKK14*) had 24 exons, which were consistent with the exon numbers of their orthologs in Arabidopsis and rice. All members from Raf and ZIK possessed 2–17 exons and 7–9 exons respectively. This conserved exon numbers in each subgroup among all three species supported their close evolutionary relationship and the introduced classification of subgroups.

**Figure 3:**
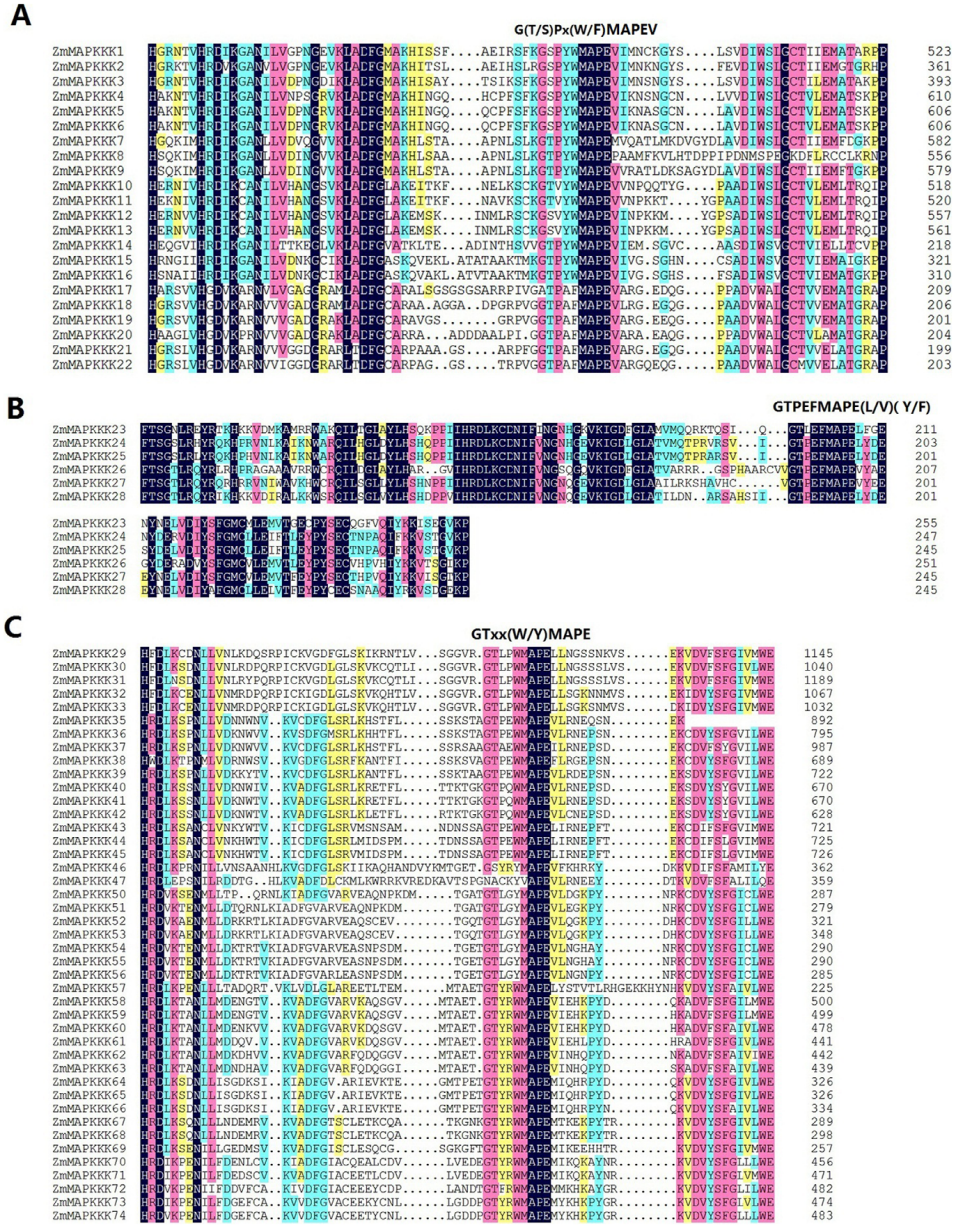
Alignment of MAPKKK family from maize. The highlighted part shows the conserved motif. A, MEKK; B, ZIK; C, Raf.

Using Clustal X to analyze the full protein sequences of all MAPKKKs, we found that the most of the Raf group proteins had a C-terminal kinase domain and extended N-terminal domains. However, most of the ZIK group members had N-terminal kinase domain whereas kinase domain of MEKK family protein were located either at N- or C-terminal or central part of the protein, which were consistent with their orthologs in Arabidopsis and rice (data not shown) [24]. In addition, we also investigated the conserved motif in their kinase domains. Among the three families MEKK family is relatively well characterized. Most MEKK-like proteins seem to participate in canonical MAP kinase cascades that activate downstream MKKs. AtMEKK1 and AtMEKK2 were shown to play important roles in plant innate immunity [28], [30], [52]. More recently, Hashimoto et al. (2012) reported that NbMAPKKKα, NbMAPKKKβ and NbMAPKKKγ functioned as positive regulators of PCD [53]. All the members of maize MEKK family shared conserved motif G (T/S) Px (W/F) MAPEV, which confirmed their association with MEKK family [24] (Figure 3A). ZIK-like kinases also known as WNK (With No lysine (K)), which have not been shown to phosphorylate MKKs in plants, are involved in internal rhythm. AtWNK1 phosphorylated the putative circadian clock component APRR3 *in vitro* and might be involved in a signal transduction cascade regulating its biological activity [54]. *AtWNK2/5/8* regulated flowering time by modulating the photoperiod pathway [55]. Recently, *OsWNK1* was found to respond differentially under various abiotic stresses and also showed rhythmic expression profile under diurnal and circadian conditions at the transcription level [56]. The conserved motif of ZIK family proteins in maize were investigated using Clustal X and as shown in Figure 3B, a conserved signature motif GTPEFMAPE (L/V) (Y/F) was found in all members [24]. Compared with ZIK and MEKK like families, Raf family has many more members. Two of the best-studied Arabidopsis Raf-like MAPKKKs, CTR1 and EDR1 are known to participate in ethylene-mediated signaling and defense responses. However, neither CTR1 nor EDR1 have been confirmed to participate in a classic MAPK cascade. As shown in Figure 3C, all the members of Raf family have the conserved motif GTXX (W/Y) MAPE except ZmMAPKKK47, which strongly supported their identity as members of Raf subfamily [24].

### Genomic Distribution and Gene Duplication

**Figure 4:**
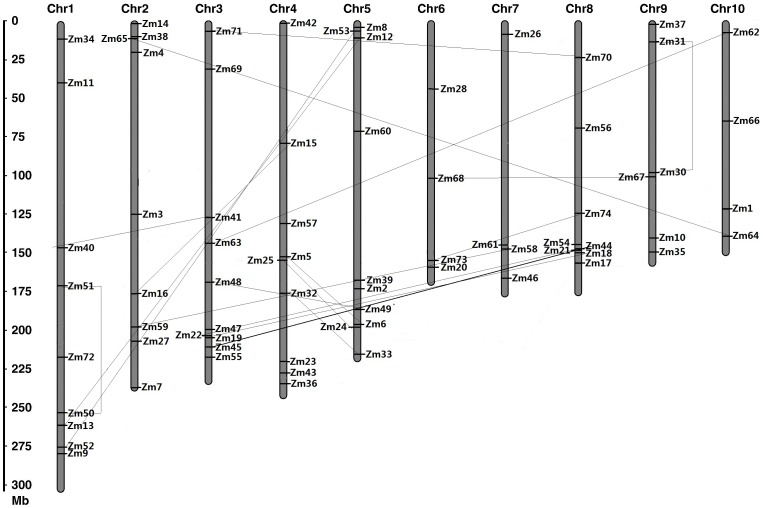
Chromosomal distributions of *MAPKKK* genes in the maize genome.

The physical locations of the MAPKKK genes on maize chromosomes were depicted in Figure 4. It was found that 73 *ZmMAPKKKs* were mapped on all 10 chromosomes of maize and 1 MAPKKK (*ZmMAPKKK29*) was situated on unanchored contigs (chromosome unknown). Ten were present on chromosomes 3 and 5; nine on chromosomes 1, 2, 4; four on chromosomes 6, 7, 10; In addition, chromosome 8 had 8 MAPKKK members, whereas chromosome 9 encoded 6 MAPKKKs members.

Gene duplication events play a significant role in the amplification of gene family members in the genome. Several rounds of genome duplication events have been found in maize genome [57]. The expansion mechanism of the maize MAPKKK gene family was analyzed to understand gene duplication events. As shown in Figure 4, nineteen paralogs of the 74 maize MAPKKKs were identified, including 17 segmental duplication events between chromosomes and the other 2 duplication events within the same chromosome (*ZmMAPKKK30* and *ZmMAPKKK31*, *ZmMAPKKK50* and *ZmMAPKKK51*). Furthermore, these gene pairs shared similar exon-intron structures. This result suggested the duplication events play vital roles in MAPKKK genes expansion in maize genome.

### Expression Pattern of the Maize MAPKKK Genes in Different Tissues and Developmental Stages

**Figure 5:**
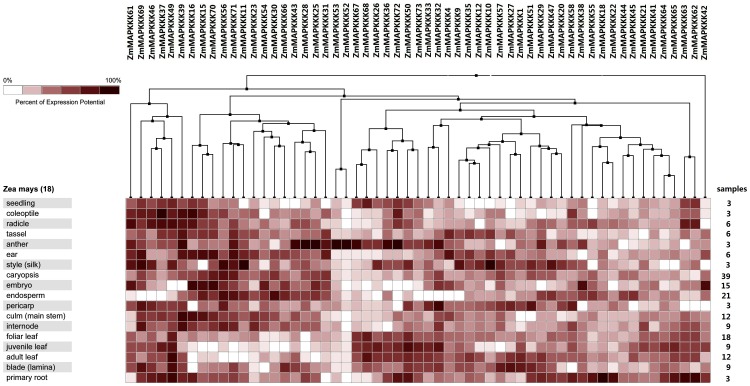
The expression profile of 57 *MAPKKK* genes in maize different tissues. The deep and light red shading represents the relative high or low expression levels, respectively.

To observe expression profiles of the MAPKKK in maize development, we analyzed the expression of the MAPKKK genes under normal growth conditions by a Genevestigator analysis (https://www.genevestigator.ethz.ch/) in 18 different tissues, including the seedlings, coleoptiles, radicles, tassel, anther, ear, silk, caryopsis, embryo, endosperm, pericarp, culm, internode, foliar leaf, juvenile leaf, adult leaf, blade and primary root. Fifty seven genes correspond to probes and there were 17 MAPKKK genes whose corresponding probes were not found. Heatmap representation of expression profile of 57 MAPKKK genes during maize development was shown in Figure 5. Eight MAPKKKs (*ZmMAPKKK25*, *ZmMAPKKK28*, *ZmMAPKKK36*, *ZmMAPKKK43*, *ZmMAPKKK52*, *ZmMAPKKK53*, *ZmMAPKKK67* and *ZmMAPKKK72*) had higher expression in anther than that of in other organs. Eight MAPKKKs (*ZmMAPKKK4*, *ZmMAPKKK9*, *ZmMAPKKK32*, *ZmMAPKKK33*, *ZmMAPKKK37*, *ZmMAPKKK46*, *ZmMAPKKK69* and *ZmMAPKKK73*) had higher expression in embryo than that of in endosperm, whereas *ZmMAPKKK22*, *ZmMAPKKK29*, *ZmMAPKKK39* and *ZmMAPKKK49* had the opposite expression profiles in embryo and endosperm. In addition, five MAPKKKs (*ZmMAPKKK18*, *ZmMAPKKK22*, *ZmMAPKKK55*, *ZmMAPKKK63* and *ZmMAPKKK62*) were expressed with high abundance in primary roots which was consistent with their expression in radicle. Specifically, *ZmMAPKKK10* and *ZmMAPKKK11* demonstrated a unique expression pattern in silk. Furthermore, MAPKKK duplicated gene pair expression patterns were also investigated, only seven pairs (*ZmMAPKKK33* and *ZmMAPKKK32*, *ZmMAPKKK44* and *ZmMAPKKK45*, *ZmMAPKKK52* and *ZmMAPKKK53*, *ZmMAPKKK64* and *ZmMAPKKK65*, *ZmMAPKKK63* and *ZmMAPKKK62*, *ZmMAPKKK67* and *ZmMAPKKK68*, *ZmMAPKKK70* and *ZmMAPKKK71*) shared the similar expression patterns in nearly all the organs, whereas other paralogs were not the case. These results showed that although the duplicated genes had higher similarities in amino acid, they may not have similar function or are involved in the same signaling pathway.

**Figure 6:**
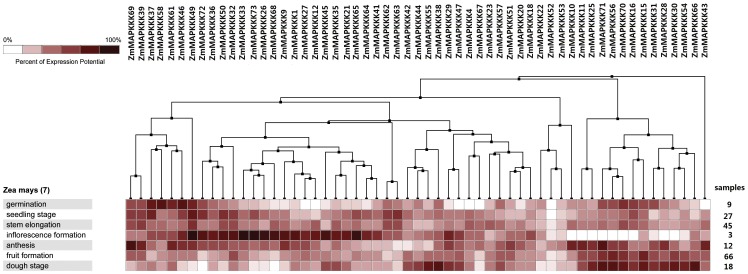
The expression profile of 57 *MAPKKK* genes in maize developmental stages. The deep and light red shading represents the relative high or low expression levels, respectively.

In addition, we also identified the expression profiles of MAPKKK family genes under different developmental stages through analysis of publicly available microarray data sets. All the 57 genes were expressed in at least one of developmental stages (Figure 6). Nine MAPKKK genes (*ZmMAPKKK10*, *ZmMAPKKK11*, *ZmMAPKKK16*, *ZmMAPKKK25*, *ZmMAPKKK28*, *ZmMAPKKK30*, *ZmMAPKKK56*, *ZmMAPKKK70*, *ZmMAPKKK71*) were expressed in all developmental stages mentioned in the Figure 6 except for inflorescence formation stage, whereas another nine MAPKKK genes (*ZmMAPKKK1*, *ZmMAPKKK9*, *ZmMAPKKK26*, *ZmMAPKKK33*, *ZmMAPKKK45*, *ZmMAPKKK49*, *ZmMAPKKK65*, *ZmMAPKKK68*, *ZmMAPKKK73*) had higher expression in inflorescence formation than that of in other developmental stages. In addition, *ZmMAPKKK37*, *ZmMAPKKK46* and *ZmMAPKKK58* had higher expression in germination stage than other genes, whereas *ZmMAPKKK43*, *ZmMAPKKK69* and *ZmMAPKKK71* had highest expression in anthesis stage. Specifically, *ZmMAPKKK52* was expressed with low abundance in all stages. Moreover, several paralogs (*ZmMAPKKK15* and *ZmMAPKKK16*, *ZmMAPKKK71* and *ZmMAPKKK70*, *ZmMAPKKK52* and *ZmMAPKKK53*, *ZmMAPKKK62* and *ZmMAPKKK63*, *ZmMAPKKK64* and *ZmMAPKKK65*) showed highly similar expression profiles, which may indicate subfunctionalization in the course of evolution. However, other gene pairs showed quit different under the maize developmental stages.

**Figure 7:**
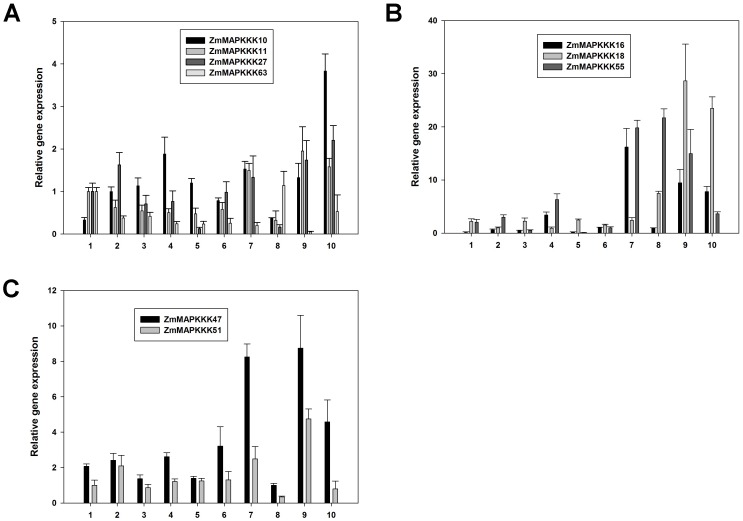
Expression patterns of the 9 *MAPKKK* genes in various tissues by quantitative real-time RT-PCR analysis. Error bars indicate standard deviations (n = 3). 1, primary root; 2, pericarp; 3, internode; 4, adult leaf; 5, silk; 6, culm; 7, seedling; 8, endosperm; 9, embryo; 10, tassel.

Next, we used quantitative real-time RT-PCR to validate the expression patterns in different tissues resulting from microarray database. Nine genes (*ZmMAPKKK10*, *ZmMAPKKK11*, *ZmMAPKKK16*, *ZmMAPKKK18*, *ZmMAPKKK27*, *ZmMAPKKK47*, *ZmMAPKKK51*, *ZmMAPKKK55*, and *ZmMAPKKK63*) were selected to confirm their expression in primary root, pericarp, internode, adult leaf, silk, culm, seedling, endosperm, embryo and tassel. Surprisingly, most our qRT-PCR data did not correspond with microarray data (Figures 5 and 7). For example, our qRT-PCR results showed that *ZmMAPKKK11*, *ZmMAPKKK18*, *ZmMAPKKK47* and *ZmMAPKKK51* exhibited a highest expression level in embryo (Figure 7), and the microarray data showed that these four genes had higher expression in silk, root and coleoptiles than that of in embryo (Figure 5). However, *ZmMAPKKK11*, *ZmMAPKKK18*, *ZmMAPKKK47* and *ZmMAPKKK51* showed higher expression in dough stage (Figure 6), suggesting they may play important roles in seed development and which was consistent with our qRT-PCR data (Figure 7). The conflicting results between our qRT-PCR and microarray database may be due to the different plant materials and growth conditions, and different experimental conditions. From these results, it is speculated that most of MAPKKK genes with different expression levels in all the maize detected organs might play key roles in plant development and several MAPKKK genes may uniquely function in maize developmental stages. However, more researches are needed to determine the functions of the MAPKKK family by additional biological experiments.

### Conclusion

An increasing body of evidence has shown that the mitogen-activated protein kinase (MAPK) cascades are involved in plant development and stress responses. So far, MAPKKKs have been investigated in several plant species including Arabidopsis and rice, no systematic analysis has been conducted in maize. In this present study, we performed a genome-wide survey and identified 74 MAPKKK genes from maize. Phylogenetic analysis of MAPKKKs from maize, rice and Arabidopsis has classified them into three subgroups. Members within each subgroup may have recent common evolutionary origins since they shared conserved protein motifs and exon-intron structures. Furthermore, microarray analysis showed that a number of maize MAPKKK genes differentially expressed across different tissues and developmental stages. In addition, quantitative real-time RT-PCR was performed on nine selected MAPKKK genes to confirm their expression patterns in different tissues. Our observations may lay the foundation for future functional analysis of maize MAPKKK genes to unravel their biological roles.

## Supporting Information

File S1 Supporting Information file contains Figure S1 and Table S1.
(DOC)
