# Supplementary material for: Genome-Wide Identification and Analysis of Expression Profiles of Maize Mitogen-Activated Protein Kinase Kinase Kinase
Source: PLoS One. 2013 Feb 27;8(2):e57714. doi: 10.1371/journal.pone.0057714 (PMC3584077; doi:10.1371/journal.pone.0057714)
Supplement: File S1 — Supporting Information file contains Figure S1 and Table S1. (DOC) [file pone.0057714.s001.doc]

**Figure S1** Phylogenetic tree of maize MAPKKKs. Neighbor-joining tree was created using MEGA5.0 program with 1,000 bootstrap using full length sequences of 74 maize. Red boxes: duplicated genes.


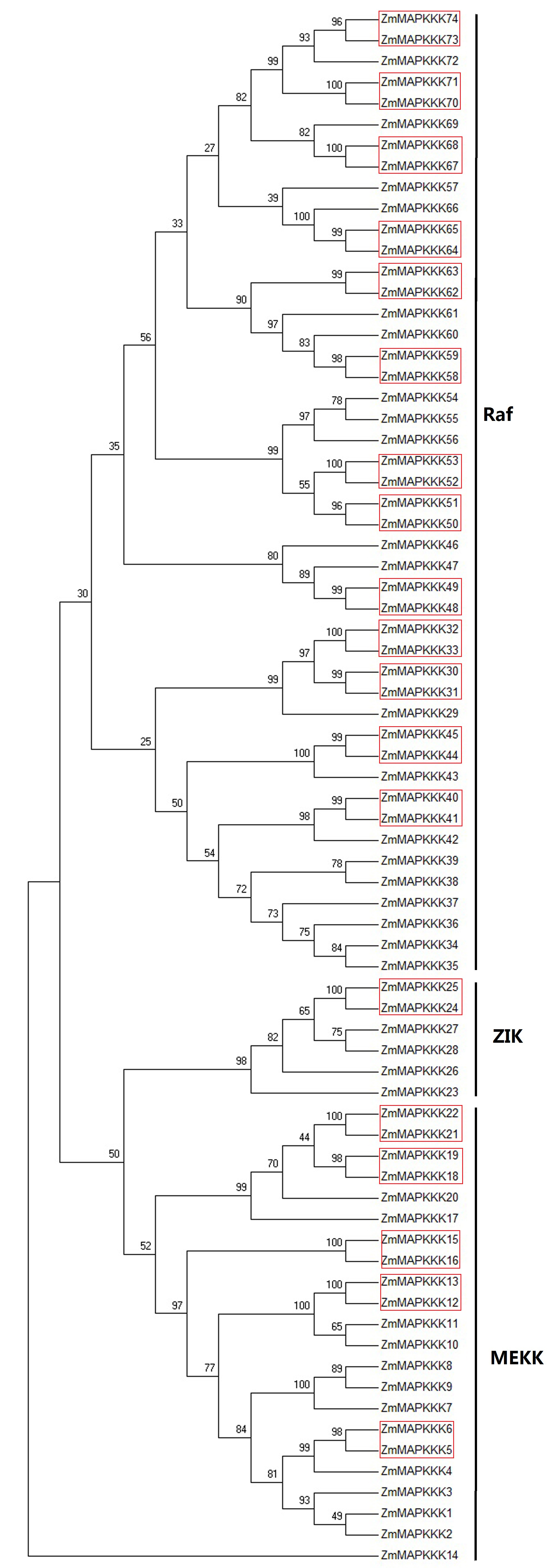


**Table S1** The primer sequences used for qRT-PCR amplification

| Gene | Forward primer | Reverse primer |
| --- | --- | --- |
| *ZmMKKK10* | 5'CACCTCAAGCACCACCACAG 3' | 5' GTCCCAAACGAGCCACTACC 3' |
| *ZmMKKK11* | 5' AGACCTCAAGCACCACCACT 3' | 5' TGTCCCAAACGAGCCACTAC 3' |
| *ZmMKKK16* | 5'GGAACTTGAGAGGGAGAGAG3' | 5' GGGATGAAATAAAGAGACGA 3' |
| *ZmMKKK18* | 5' ACTGGGATTCCGAAGAAGGC 3' | 5' ACTCAGGATCAGGAGCTGGC 3' |
| *ZmMKKK27* | 5'GGCTGAGCTGTTTGTAAATT 3' | 5' TAGAGTCCCATGCCTTGTTT 3' |
| *ZmMKKK47* | 5'GATTGAACAGTGCTGGAGCG 3' | 5' TGAGCGTGATGAACGGGTGG 3' |
| *ZmMKKK51* | 5' CTCTACTTGTCTCTGCCCTA 3' | 5' CTCACTGGTATTCACATCAC 3' |
| *ZmMKKK55* | 5'CAGTACCGAGGTCCCTAACG 3' | 5' AGCATACAAGCACACCAATC 3' |
| *ZmMKKK63* | 5' GTAAGGCAGGACACACCATA 3' | 5' CCATCAACAACGAAAACATC 3' |
| *Zmactin* | 5' ATCCAGGCTGTTCTTTCGTT 3' | 5' CATTAGGTGGTCGGTGAGGT 3' |
